# Molecular Characterization of 4/91 Infectious Bronchitis Virus Leading to Studies of Pathogenesis and Host Responses in Laying Hens

**DOI:** 10.3390/pathogens10050624

**Published:** 2021-05-19

**Authors:** Shahnas M. Najimudeen, Mohamed S. H. Hassan, Dayna Goldsmith, Davor Ojkic, Susan C. Cork, Martine Boulianne, Mohamed Faizal Abdul-Careem

**Affiliations:** 1 Department of Ecosystem and Public Health, Faculty of Veterinary Medicine, University of Calgary, Calgary, AB T2N 4N1, Canada; fathimashahnas.moham@ucalgary.ca (S.M.N.); msh.hassan@ucalgary.ca (M.S.H.H.); dayna.goldsmith@ucalgary.ca (D.G.); sccork@ucalgary.ca (S.C.C.); 2 Department of Poultry Diseases, Faculty of Veterinary Medicine, Assiut University, Assiut 71515, Egypt; 3 Animal Health Laboratory, University of Guelph, Guelph, ON N1G 2W1, Canada; dojkic@uoguelph.ca; 4 Department of Clinical Sciences, Faculty of Veterinary Medicine, University of Montréal, St. Hyacinthe, QC J2S 2M2, Canada; martine.boulianne@umontreal.ca

**Keywords:** infectious bronchitis virus, pathogenesis, tissue tropism, reproductive tract, laying hens, immunofluorescence assay

## Abstract

Infectious bronchitis virus (IBV) initially establishes the infection in the respiratory tract and then spreads to other tissues depending on its virulence. During 2011–2018, the 4/91 IBV strain was isolated from poultry flocks affected by decreased egg production and quality in Eastern Canada. One of the Canadian 4/91 IBV isolates, IBV/Ck/Can/17-038913, was propagated in embryonated chicken eggs and molecularly characterized using whole genome sequencing. An in vivo study in laying hens was conducted to observe if IBV/Ck/Can/17-038913 isolate affects the egg production and quality. Hens were infected with IBV/Ck/Can/17-038913 isolate during the peak of egg lay, using a standard dose and routes maintaining uninfected controls. Oropharyngeal and cloacal swabs were collected at predetermined time points for the quantification of IBV genome loads. At 6 and 10 days post-infection, hens were euthanized to observe the lesions in various organs and collect blood and tissue samples for the quantification of antibody response and IBV genome loads, respectively. Egg production was not impacted during the first 10 days following infection. No gross lesions were observed in the tissues of the infected birds. The IBV genome was quantified in swabs, trachea, lung, proventriculus, cecal tonsils, kidney, and reproductive tissues. The serum antibody response against IBV was quantified in infected hens. In addition, histological changes, and recruitment of immune cells, such as macrophages and T cell subsets in kidney tissues, were measured. Overall, data show that IBV/Ck/Can/17-038913 isolate is not associated with egg production issues in laying hens infected at the peak of lay, while it demonstrates various tissue tropism, including kidney, where histopathological lesions and immune cell recruitments were evident.

## 1. Introduction

Infectious bronchitis (IB) is a multi-system disease in chickens caused by infectious bronchitis virus (IBV), which is a *Gammacoronavirus* that belongs to the family *Coronaviridae* and order *Nidovirales* [1]. The disease was first recorded in the United States in the early 1930s [2]. Since then, outbreaks of IB in layer and broiler flocks have been reported globally with considerable economic losses due to poor growth performances in broilers, and egg production and quality defects in layers [3]. Although vaccine-mediated control measures are extensively employed, IB is still a major issue in the poultry industry in Canada and globally [4,5,6,7,8]. 

IBV is a positive-sense RNA virus with a genome of around 27.6 kilobase pairs (kb) length [9]. Of the *IBV* genes, the replicase gene occupies the 5’ two-thirds of the genome (~20 kb) and expresses two polyproteins (1a and 1ab) using a mechanism known as −1 ribosomal frameshift. With the help of two virus-encoded proteases, the two polyproteins are post-translationally divided into nonstructural proteins (nsps, n = 15) that function in the replication and pathogenicity of the virus [10]. The remaining one-third (~7 kb) of the 3’ end of the genome translates into four structural proteins: spike glycoprotein (S), the membrane glycoprotein (M), the phosphorylated nucleocapsid protein (N) and the small envelope protein (E). Additionally, some accessory proteins that vary in number and position among different IBV strains are encoded by the IBV genome [11]. According to the previous description, the genome of IBV is arranged in the order, 5′-1a-1ab-S-3a-3b-E-M-5a-5b-N-3′ with 5 and 3 genes encoding for accessory proteins that have unclear functions [12]. The 5’ and 3’ ends of the genome are flanked by short sequences (~0.5 kb) of untranslated regions (UTR) that have regulatory elements required for RNA replication and transcription [13].

IBV enters the host via inhalation and then replicates in the ciliated epithelial lining and mucus-secreting glands of the respiratory tract [14]. The lesions caused by IBV in the trachea, nasal passage, and sinuses cause accumulation of catarrhal, serous, or caseous exudates [15] leading to clinical manifestations such as sneezing, gasping, coughing, tracheal rales, nasal discharge, and dyspnoea [16]. Moreover, IBV can travel from the respiratory tract via the bloodstream and the monocytes [17], and, based on the genotype, infects the kidneys, reproductive tract, gastrointestinal tract, cecal tonsils, proventriculus, and muscle tissues [18,19,20,21,22,23]. The persistence of the virus and subsequent pathology in these organs also vary with the infecting IBV strain and host factors such as age, immunity, and genetics [18,24,25]. The persistence of the virus in the kidneys and cecal tonsils is longer when compared to other tissues [16]. The nephropathogenic IBV strains, such as QX-like, Aust T and B1648 lead to severe lesions in the kidneys [17,26,27,28], particularly in young birds, leading to high mortality [26,29]. The infected kidneys grossly appear pale in color with a mottled surface and distended tubules with urate deposits [17,18]. Although bilateral pectoral myopathy due to IBV infection is not recorded in North America, some European strains such as 793/B could lead to this condition [20]. Bilateral pectoral myopathy is characterized by edema and hemorrhages of pectoral muscles [21]. 

The M41, Aust T and QX-like IBV strains are linked to reproductive tract disease in laying hens [24,30] that range from mild discoloration of the eggs to severe production failure with poor internal and external egg quality [31]. The production drop may range from 35–63% [32]. Infection in chickens less than two weeks old with these IBV strains has been shown to cause cystic oviduct, leading to false layer syndrome [19,24,27,32,33].

The 4/91 IBV strain was first isolated in France in the mid-1980s and, currently, shows a global distribution except for the USA and Australia [34]. The virus is 96% similar in the *S1* gene to the Moroccan IBV G strain [35]. When compared to other IBV strains, 4/91 IBV has a very broad tissue tropism towards the respiratory tract, kidneys [36], gastrointestinal tract [21], and pectoral muscles [20]. The Animal Health Laboratory, University of Guelph, Guelph, Ontario, reported that the 4/91 strain has been one of the common IBV strains isolated in Eastern Canada between 2014 and 2018 from laying flocks that recorded poor egg production [4]. However, the detailed molecular nature of 4/91 IBV circulating in Canada and its pathogenesis in laying hens are not known. The information is also scarce on host responses mounted against 4/91 IBV. The present study was designed to (1) molecularly characterize 4/91 IBV isolated from poultry flocks in Canada and (2) investigate the pathogenesis and elicited host responses of experimental 4/91 IBV infection in laying hens.

## 2. Results

### 2.1. Phylogenetic Analysis and Full Genome Characterization

**Figure 1:**
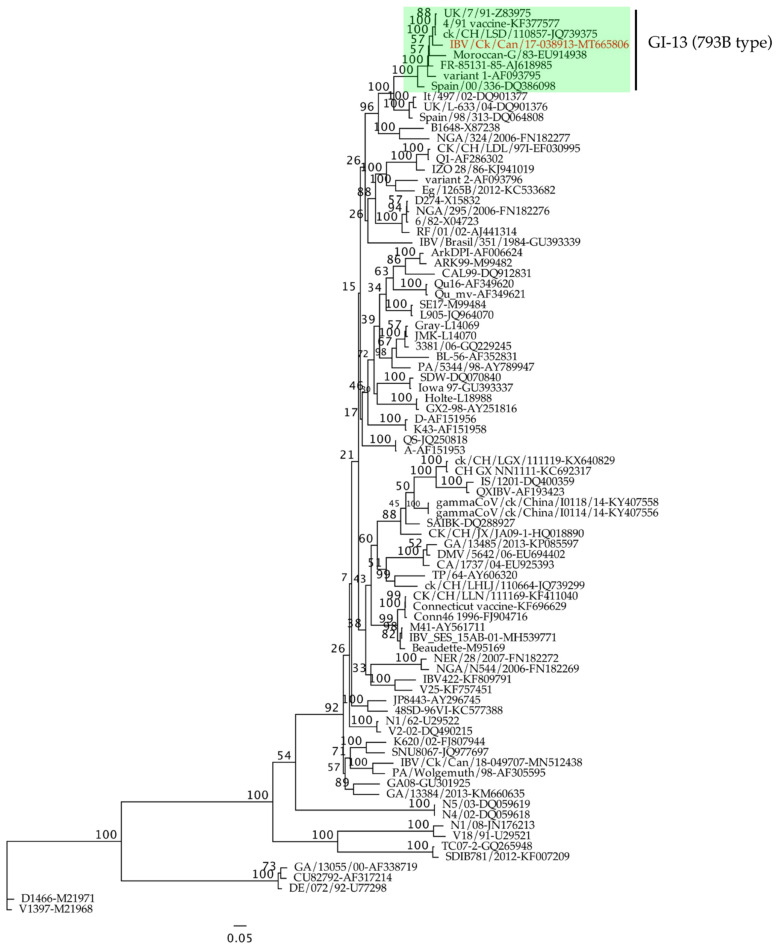
Randomized accelerated maximum likelihood (RAxML) phylogenetic tree based on the complete nucleotide sequences of *S1* genes of the Canadian 4/91 IBV isolate, IBV/Ck/Can/17-038913 (marked in red color) and 86 reference sequences.

The general time-reversible model with gamma distribution and 1000 bootstrap replicates was used for the construction of a maximum likelihood phylogenetic tree of the 87 complete *S1* genes. IBV/Ck/Can/17-038913 isolate clustered within the GI-13 lineage along with IBVs isolated from different parts around the world that are known as 793B type (also known as 4/91 and CR88; Figure 1). The complete genome of IBV/Ck/Can/17-038913 isolate consists of 27,472 nucleotides, excluding the poly (A) tail. Twelve ORFs (5′-1a-1b-S-3a-3b-E-M-4b-4c-5a-5b-N-3′) were predicted (Table 1). 

**Figure 2:**
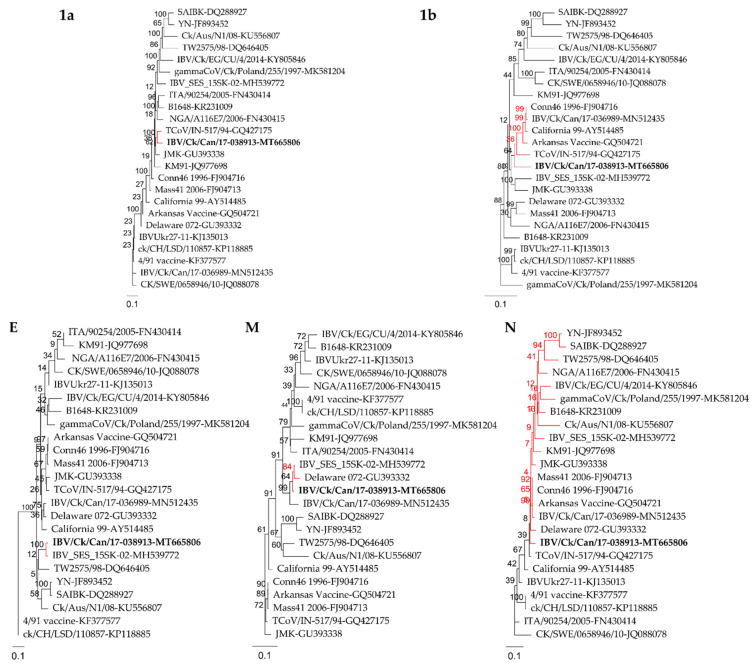
Randomized accelerated maximum likelihood (RAxML) phylogenetic trees based on the nucleotide sequences of *1a*, *1b*, *E*, *M*, and *N* genes of the Canadian 4/91 IBV isolate, IBV/Ck/Can/17-038913 (marked in bold font) and 23 IBV reference sequences. IBV/Ck/Can/17-038913 isolate cluster is shown in red color.

Sequence comparison analysis demonstrated that the genomic nucleotide identity between IBV/Ck/Can/17-038913 isolate and different IBV strains ranged from 86.1% (TW2575/98) to 92.0% (JMK) while it showed 87.2% identity with turkey coronavirus (TCoV) isolate (TCoV/IN-517/94). Among the different ORFs of the genome, the *S* gene was the most variable (70.9–96.7%), whereas it showed only 48.3% identity with TCoV (TCoV/IN-517/94). On the other hand, the 5b accessory protein was the most conserved (91.6–98.4%). IBV/Ck/Can/17-038913 isolate revealed higher sequence identities (93.3–99.0%) of the 3b, E, M, 4b, 4c, 5a and 5b ORFs with the Canadian Massachusetts (Mass) type isolate (IBV_SES_15SK-02). The 3a and N had the highest nucleotide identity with the Conn46 1996 strain at 94.8% and 96.7%, respectively. ORF 1a had the highest nucleotide identity (93.5%) with TCoV/IN-517/94. The IBVs of 793B type, 4/91 vaccine, ck/CH/LSD/110857 and IBVUkr27-11, showed the highest identity (95.1–96.7%) with the *S* gene (Table 2). Phylogenetic trees constructed based on different genomic regions showed different topologies and confirmed the results of the sequence comparison. The IBV/Ck/Can/17-038913 isolate did not cluster with 793B type strains outside of the *S1* gene, whereas it clustered with the Mass type (*1a* and *E* genes) or Mass and non-Mass type strains (*1b*, *M* and *N* genes; Figure 2).

### 2.2. Recombination Analysis

**Figure 3:**
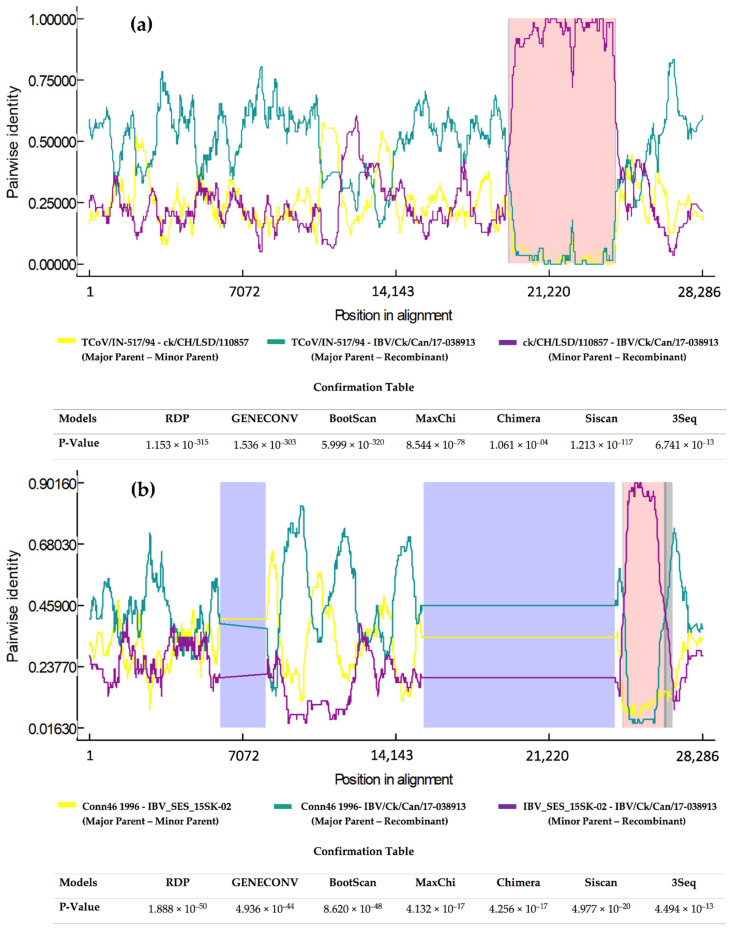
RDP4 recombination plot and confirmation table of the recombination events associated with IBV/Ck/Can/17-038913 isolate. Putative recombination region is shaded in pink color. (**a**) Breakpoint 19,284–23,701 nucleotides; (**b**) Breakpoint 24,125–25,927 nucleotides.

The recombination analysis identified two potential recombination events by the seven models employed by Recombination Detection Program (RDP) 4 software. The first recombinant region is located between 19,284 and 23,701 nucleotides that are found in the extreme 3′ end of the replicase gene and the *S* gene. In this case, the minor parent belongs to the ck/CH/LSD/110857 strain (KP118885), and the major parent to the TCoV/IN-517/94 strain (GQ427175; Figure 3a). The second recombinant region located between 24,125 and 25,927 nucleotides, containing *E*, *4* and *5* genes with a minor parent, was inferred as the IBV_SES_15SK-02 strain (MH539772), and the putative major parent was determined as the Conn46 1996 strain (FJ904716; Figure 3b).

### 2.3. Embryo Lesions

Embryo passage resulted in IBV induced lesions such as stunting and curling.

### 2.4. Clinical and Pathological Manifestations in Laying Hens

A significant drop in egg production or changes in egg quality, such as egg shape, albumin thickness and eggshell structure, compared to the uninfected control group, were not observed during the study period (*p* = 0.8630; Supplementary Figure S1). No clinical signs were observed in the infected or the control group. At the necropsy examination performed at 6 and 10 dpi, no gross lesions were noted in any tissues of the infected or uninfected control hens.

### 2.5. IBV Genome Loads in Oropharyngeal, Cloacal Swabs and Tissue Samples

**Figure 4:**
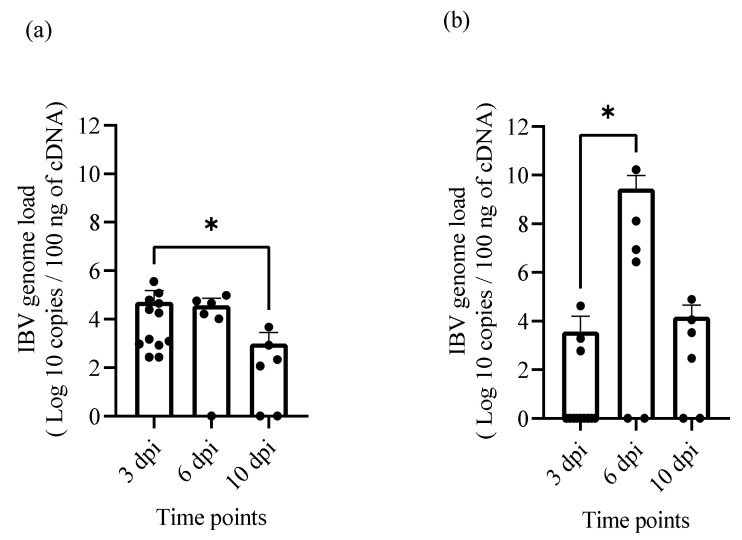

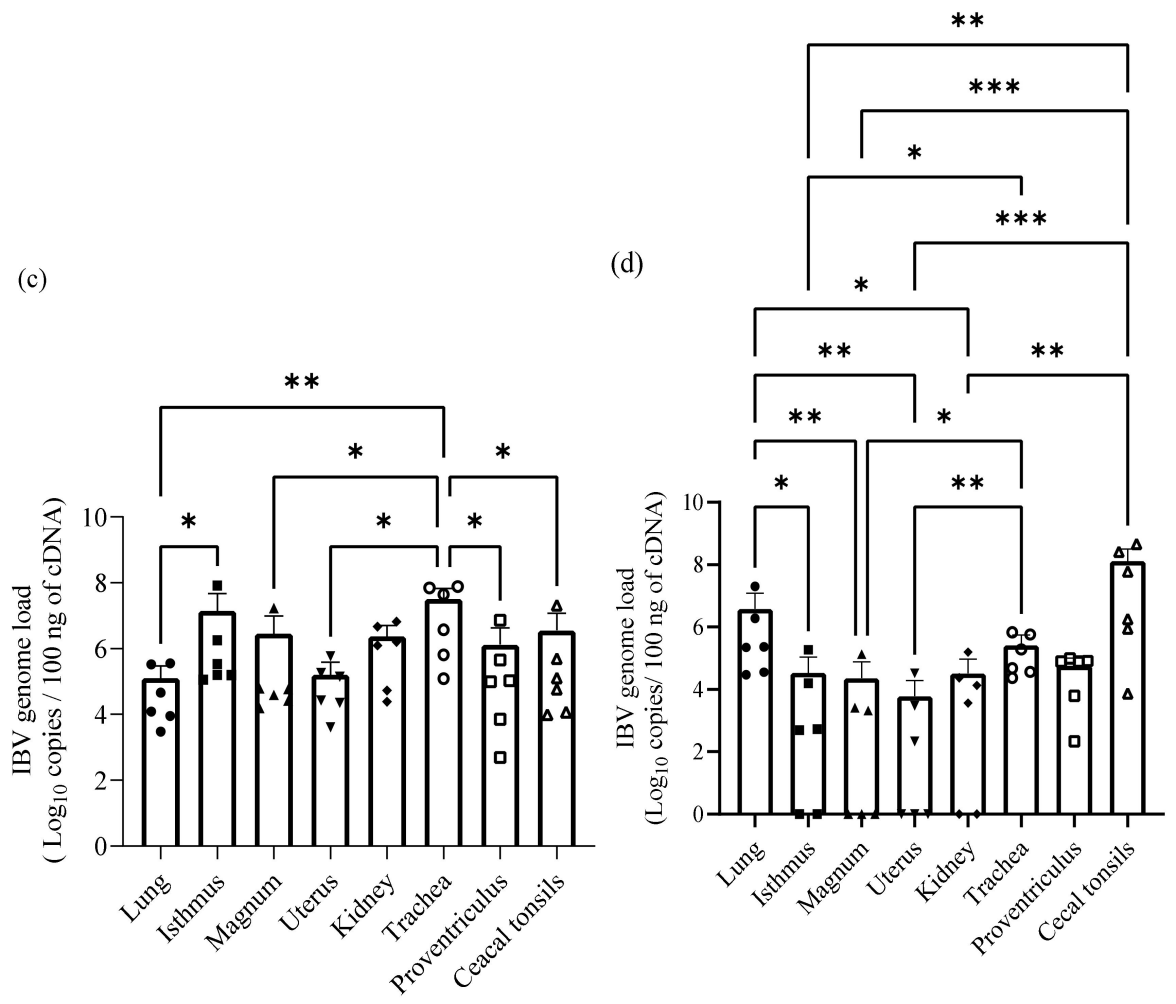
IBV genome loads in swabs and tissues following IBV/Ck/Can/17-038913 isolate infection. The mean IBV genome loads in 100 ng of extracted RNA from OP swabs (**a**), CL swabs (**b**) at 3, 6, 10 dpi and from various tissues of infected hens at 6 dpi (**c**) and 10 dpi (**d**) are shown here. The IBV genome loads in swabs and tissues were compared using the Kruskal–Wallis test followed by Dunn’s multiple comparison. The error bars represent the SD. Significance: * *p* < 0.05, ** *p* < 0.01, *** *p* < 0.001.

Viral genome loads in oropharyngeal (OP) and cloacal (CL) swabs and tissue samples including trachea, lung, kidney, cecal tonsils, proventriculus, and reproductive tract (uterus, magnum and isthmus) in the infected group were quantified using SYBR Green qRT-PCR assay. A significant difference in the IBV genome load was observed between 3 and 10 dpi (*p* = 0.0452) in OP swabs (Figure 4a) and 3 and 6 dpi in CL swabs (*p* = 0.0472; Figure 4b). The viral loads in the tissues of different organs at 6 and 10 dpi are shown in Figure 4c,d, respectively. At 6 dpi, the highest genome load was found in the trachea and isthmus, respectively, while the lowest amount was found in the lung. However, the highest viral load at 10 dpi was observed in the cecal tonsils, which is an organ of IBV persistence, followed by the lung. In contrast, the reproductive tract had the lowest amount of IBV genome load compared to other tissues.

### 2.6. Host Responses

#### 2.6.1. Serum Anti-IBV Antibody (Ab) Response

**Figure 5:**
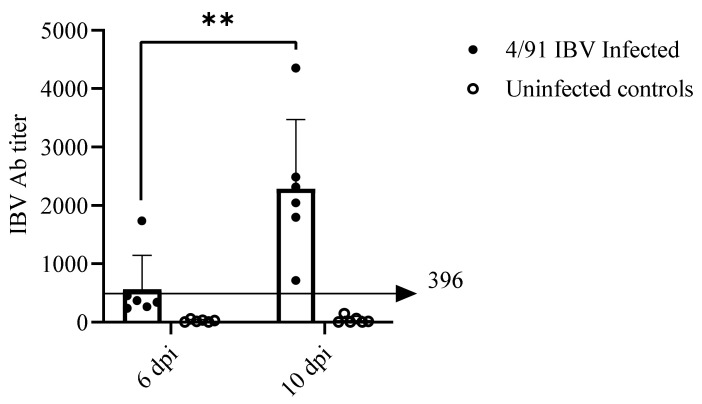
Anti-IBV Ab titers in serum samples measured using IDEXX ELISA coated with the virus particles, following IBV/Ck/Can/17-038913 isolate infection. Serum anti-IBV Ab titers of the infected group at 6 and 10 dpi were compared using the Mann–Whitney U test. The solid line at 396 indicates the cut-off value of IDEXX IBV ELISA. The error bars represent the SD. Significance: ** *p* < 0.01.

All infected hens developed anti-IBV antibody (Ab) at 10 dpi and two birds were positive at 6 dpi, whereas the hens from the control group remained negative at the two time points. A significant difference in anti-IBV Ab titer was observed between sera collected from the infected group at 6 and 10 dpi (*p* = 0.0043; Figure 5).

#### 2.6.2. Recruitment of CD4^+^ T Cells, CD8^+^ T Cells and Macrophages

**Figure 6:**
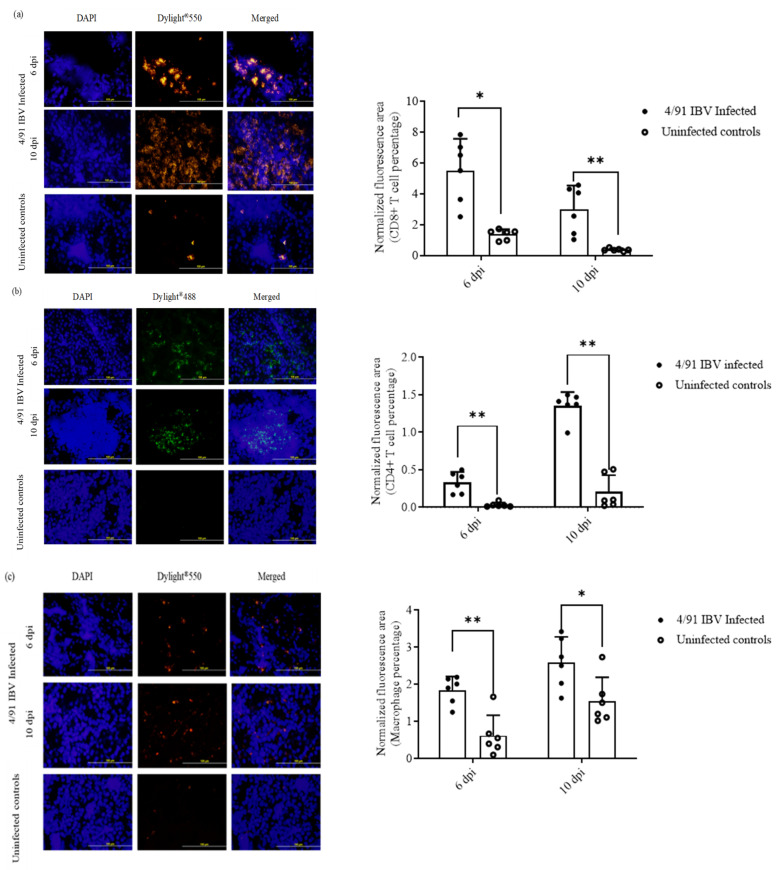
Increased number of immune cells in birds infected with IBV/Ck/Can/17-038913 isolate. The representative images of CD8^+^ T cells (**a**), CD4^+^ T cells (**b**) and macrophages (**c**) observed in the kidney tissues of the infected and uninfected hens along with quantitative data are illustrated. Comparisons between infected and uninfected groups at 6 dpi and 10 dpi were performed using the Mann–Whitney U test. The error bars represent the SD. Significance: * *p* < 0.05, ** *p* < 0.01.

A significant recruitment of CD8^+^ T cells, CD4^+^ T cells and macrophages at both 6 and 10 dpi was observed in the kidney tissues of the infected hens when compared to uninfected controls (Figure 6a–c). At both 6 and 10 dpi time points, the number of CD8^+^ cells exceeded that of CD4^+^ cells.

#### 2.6.3. Histopathological Findings in the Kidney

**Figure 7:**
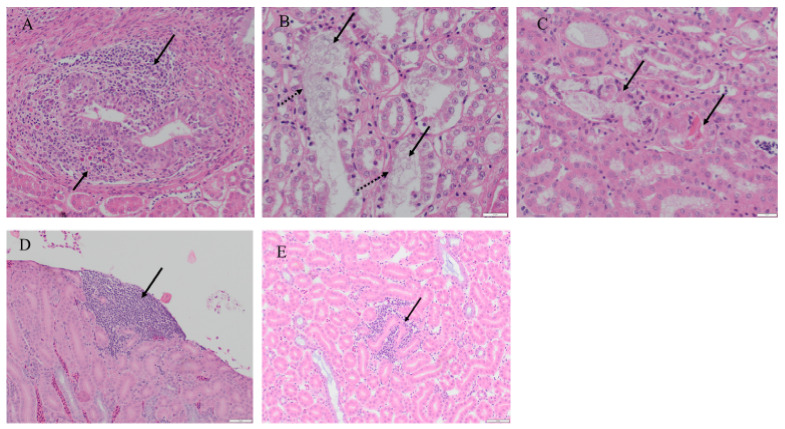
Histopathological changes in kidney tissues induced by IBV/Ck/Can/17-038913 isolate infection. The representative images show (**A**) infiltration of the inflammatory cells, (**B**) dilation of the kidney tubules (solid arrow) and urate crystals (dotted arrow), (**C**) tubular epithelial necrosis in 10 dpi infected kidney tissues and (**D**), (**E**) mild lymphoid aggregates in 6 dpi infected and control tissues, respectively.

Although no gross lesions were observed during necropsy examination, histological changes were present in renal tissues of the infected hens at 10 dpi with no significant changes at 6 dpi (Figure 7). Mild to moderate dilation of collecting ducts was observed with an increased luminal accumulation of urate spherules in all infected birds at 10 dpi (Table 3). The dilated tubules were most prominent in the medullary area with occasional sloughing of epithelial cells and rare luminal hyper eosinophilic necrotic cellular debris. Mononuclear cell infiltration was observed in two birds of the infected group. Around the collecting ducts and ureteral branches, the subepithelial connective tissue was infiltrated by moderate numbers of lymphocytes and macrophages along with fewer plasma cells and leukocytes with large eosinophilic cytoplasmic vacuoles (suspected Mott cells). Small numbers of lymphocytes were observed extending into the lining epithelium and there was rare associated karyorrhectic debris. Mild collecting duct dilation was seen in one uninfected hen and no other notable histological changes were observed among uninfected kidney tissues. No significant histologic abnormalities within any of the examined kidney sections (infected and control) were seen at 6 dpi.

## 3. Discussion

The recovery of a significant percentage of 4/91 IBV isolates from Eastern Canadian poultry flocks, which had problems with egg production in the recent past [4], led us to investigate the impact of Canadian 4/91 IBV infection in laying hens. The present study yielded several findings. First, based on *S1* gene phylogenetic analysis, our IBV/Ck/Can/17-038913 isolate grouped within the GI-13 lineage along with 793B IBVs (also known as 4/91 and CR88). Second, the IBV/Ck/Can/17-038913 isolate leads to subclinical infection in laying hens with no impact on egg production. Third, the IBV genome load data suggest that IBV/Ck/Can/17-038913 isolate has a very broad tissue tropism. Fourth, when we focused our investigation on the kidney, we found that IBV/Ck/Can/17-038913 isolate induces histological changes in the kidney with significant immune cell recruitment.

In Canada, several IBV variants were detected in Canadian poultry operations over the last two decades [4,37]. However, the published data that involved detailed molecular characterization of different Canadian IBV strains are scarce. Amongst the IBV strains isolated in Canada, there are only seven whole genome sequences available through GenBank [38,39]. Hence, in the present study, the complete genome of the Canadian IBV isolate, IBV/Ck/Can/17-038913, was characterized to further understand the genetic backgrounds of IBV evolution in Canada. The results of comparative genomics showed high nucleotide identity between our isolate and TCoV/IN-517/94 in the replicase gene. This finding was not surprising, whereas this TCoV was shown to be originated as a consequence of recombination that swapped the spike gene of a Mass IBV strain [40]. Additionally, another Mass type isolate, IBV-SES-15SK-02, shared high nucleotide sequence identities with our isolate in genes *4* and *5* and most of gene *3*. The role of Mass type strains in the evolution of our isolate was further elucidated by identifying IBV-SES-15SK-02 isolate as a minor parent for a recombinant region in the 3′ end of the genome. Over the last two decades, a marked proportion of the IBVs detected in the Canadian poultry operations has shown to be of Mass type [4,37]. Despite the high similarity of different genomic fragments with Mass type strains, another recombination event occurred during the origin and evolution of the IBV/Ck/Can/17-038913 isolate and caused it to cluster with the 793/B group. Within the *S* gene, natural recombination events associated with shifts in the serotype and/or genotype of the circulating IBVs were described in the past [39,41].

The 4/91 IBV strain was introduced into Eastern Canada in mid-2011, and around 50% of IBV isolates that was detected from the flocks affected with respiratory and egg production problems during 2012–2013 were identified as 4/91 type [4]. According to Animal Health Laboratory data, the recovery of 4/91 IBVs has declined towards 2018 in Canada, although they are still a significant problem in other countries [7,27,36,42,43].

Although 4/91 IBV has shown to be a highly pathogenic virus strain causing severe clinical signs and mortality [42,44], some studies have reported only mild respiratory signs [21,45]. In the present study, we observed neither clinical signs nor gross lesions in any of the tissues throughout the experimental period. It is possible that this variant is less virulent in laying hens. However, the results could be different in field conditions as the severity of the disease is also influenced by other factors such as coinfections, breed, and immunity of the host [18,21,24,25], which are controlled in our experimental design.

Since 4/91 IBV was associated with kidney tissues [21,27,46], we focused our studies on the kidney. Although gross lesions were not observed in IBV/Ck/Can/17-038913 isolate infected kidneys, histopathological changes were seen. At 10 dpi, infected kidney tissues were comprised of tubular dilation with luminal urate accumulation, desquamation, and mononuclear cell infiltration with rare epithelial necrosis. These findings were similar to the histological lesions caused by nephropathogenic IBV strains, such as QX-like, Aust T and TW-like IBV [18,27,47]. Moreover, nephropathogenic lesions and mortality induced by nephropathogenic IBV strains are more prominent in young chickens than adults [26,29]. Therefore, our 4/91 IBV isolate may be a nephropathogenic strain and this requires to be confirmed by infecting young chickens.

In agreement with the previous literature [21,27,45], the Canadian 4/91 IBV isolate, IBV/Ck/Can/17-038913, had a broad tissue tropism, including cecal tonsils, trachea, lung, kidney, proventriculus, and reproductive tract. At 10 dpi, the highest IBV genome load was observed in cecal tonsils, which represent an organ of IBV persistence [16,43,44,48,49], followed by the trachea. In agreement with a study by Raj and Jones [21], our data show high IBV genome loads in kidney tissues at 6 dpi followed by 10 dpi.

The egg production issues caused by IBV can vary from only mild discoloration of the eggshell to severe production failure with the deterioration of egg quality [31,50]. IBV infection in laying hens can result in a 20–90% drop in egg production [51]. However, a significant drop in egg production or deterioration of the internal or external quality of the eggs were not observed in our study. A reduction in the length of the oviduct and regressed ovaries, which are features of damage caused by IBV [24,52], were also not observed in all 12 IBV/Ck/Can/17-038913 isolate infected hens.

In summary, based on *S1* gene phylogenetic analysis and comparative genomics, the IBV/Ck/Can/17-038913 isolate was assigned to a GI-13 lineage and showed varying similarities with different reference strains across its different ORFs. In laying hens, the Canadian 4/91 IBV isolate, IBV/Ck/Can/17-038913, had a very broad tissue tropism including viral replication in the reproductive tract, while it did not cause egg production or egg quality problems with the dose of infection used in the current study. However, the IBV/Ck/Can/17-038913 isolate led to histological changes in the kidney with significant recruitment of T cell subsets and macrophages. Further studies are necessary to determine the impact of IBV/Ck/Can/17-038913 isolate infection in young chickens on renal and reproductive tissues.

## 4. Materials and Methods

### 4.1. Animals, Ethics and Virus

Specific pathogen-free (SPF) eggs for virus propagation and titration and 25-weeks-old SPF layer chickens for experimental infection were purchased from the Canadian Food Inspection Agency (CFIA), Ottawa. Ethical approval for the proposed work was obtained from the Health Science Animal Care Committee (HSACC) of the University of Calgary (Protocol number: AC19-0011).

The Canadian 4/91 IBV isolate, designated as IBV/Ck/Can/17-038913, used in the present study was recovered from a tracheal swab collected from a 2-week-old layer flock presented with respiratory manifestations in Ontario, Canada. The archived swab was obtained from Animal Health Laboratory, University of Guelph, Guelph, ON, Canada. Virus isolation in 9–11-days-old SPF embryonated chicken eggs was carried out as previously described [39]. The virus was titrated using 9-days-old SPF eggs and the titer was determined to be 1 × 10^6^ EID_50_/ mL.

### 4.2. Ribonucleic Acid (RNA) Extraction and Sequencing

After three passages in embryonated chicken eggs, RNA was extracted from allantoic fluid using Trizol LS^®^ reagent (Ambion, Invitrogen Canada Inc., Burlington, ON, Canada) according to the manufacturer’s protocol. The genome was transcribed to complimentary deoxyribonucleic acid (cDNA) using random primers (High Capacity Reverse Transcription Kit, Applied Biosystems™, Invitrogen Canada Inc., Burlington, ON, Canada) according to the manufacturer’s instructions. Whole genome sequencing on the cDNA library was performed at the Faculty of Veterinary Medicine, University of Montreal, Montreal QC, Canada, using a Miseq platform (Illumina corp, San Diego, CA, USA). The genome was deposited in GenBank under the accession number MT665806.

### 4.3. Genotyping and Sequence Analysis

The *S1* gene of the current isolate, in addition to the *S1* gene sequences of 86 IBV reference strains of different genotypes (Supplementary Table S1) [53], was selected and used for phylogenetic analysis. Clustal Omega [54] and RAxML [55] plug-ins in the Geneious^®^ software v10.2.6 (https://www.geneious.com/) were used for multiple sequence alignments and phylogenetic tree construction, respectively. The coding sequences and ORFs prediction was carried out in https://www.ncbi.nlm.nih.gov/orffinder/. Both the complete genome and nucleotide sequences of different ORFs of IBV/Ck/Can/17-038913 isolate were used for BLAST searching of the National Center for Biotechnology Information (NCBI) database and were compared to 23 reference IBV sequences of North America (USA and Canada), Asia (China, Korea and Taiwan), Africa (Nigeria and Egypt), Europe (Sweden, Poland, Ukraine, Belgium and Italy) and Australia. Phylogenetic analyses of the nucleotide sequences of *1a*, *1b*, *E*, *M* and *N* genes were also performed with the selected 23 reference IBV sequences.

### 4.4. Recombination Analysis

The complete genome sequence of the Canadian 4/91 IBV isolate, IBV/Ck/Can/17-038913 and 23 reference IBV sequences mentioned in the previous section were aligned using multiple alignments with fast Fourier transformation (MAFFT) [56] and used for recombination analysis conducted in the RDP4 (RDP v.4.101) [57]. Specific models (RDP, Genecov, Bootscan, Maxchi, Chimaera, Siscan, and 3Seq) embedded in RDP4 were used to identify putative recombination events in the genome of our isolate using default settings for most models. Changes from default settings were the window size for RDP (60) and Bootscan and SiScan (500) to reduce masking of recombination signals. Only the recombination events identified by at least five of these models were considered.

### 4.5. Experimental Infection of Laying Hens

Twenty-four 25-weeks-old SPF hens (at the peak of egg lay) were purchased from CFIA, Ottawa, and allowed to acclimatize for 19 days. Hens were placed in Animal Resource Center (ARC), Foothill campus, University of Calgary. Feed and water were provided ad libitum. The laying hens were kept under a daily light cycle of 16-h light: 8-h dark.

At the end of the acclimatization period, 12 hens were infected with IBV/Ck/Can/17-038913 isolate at a dose of 1× 10^6^ EID_50_ per chicken under isoflurane anesthesia. We used the standard dose of IBV used in adult chickens as was performed previously [58,59]. A total volume of 1 mL was inoculated via intraocular (100 µL), intranasal (100 µL) and intratracheal (800 µL) routes. The infected group was placed in high containment poultry isolators in Prion Virology facility (PVF). The remaining 12 hens were mock-infected with phosphate-buffered saline (PBS) and acted as uninfected controls. Egg production, egg quality (egg weight, shape index, albumin thickness) and clinical signs were monitored daily. Six hens from the infected and control groups were euthanized at 6 and10 days post-infection (dpi) to observe the gross lesions in various organs and to collect samples.

OP and CL swabs were collected at 3, 6 and 10 dpi into tubes containing viral transport medium (Puritan^®^UniTranz-RT^®^ Media Transport Systems, VWR, Edmonton, AB, Canada). Tissue samples of trachea, lung, cecal tonsils, kidney, oviduct (magnum, isthmus, uterus), and proventriculus were collected individually in RNA Save (Biological Industries, FroggaBio, Toronto ON, Canada), and preserved at −20 °C, for virus genome load quantification by quantitative reverse transcription-polymerase chain reaction (qRT-PCR). In addition, kidney samples were snap frozen in Optimum Cutting Temperature (OCT) medium (Tissue-Tek^®^, Sakura Finetek USA Inc., Torrance, CA, USA) for immunofluorescence studies and fixed in 10% neutral buffered formalin (VWR International, West Chester, PA, USA) for examining histological changes. Blood samples were collected into plain tubes on the day of euthanasia (6 and 10 dpi) to monitor the anti-IBV Ab response. The blood samples were kept at room temperature for 30 min, centrifuged at 2000× *g* for 10 min at 4 °C, the serum was separated, and aliquots were stored at −20 °C.

### 4.6. Techniques

#### 4.6.1. RNA Extraction and cDNA Synthesis 

The tubes containing OP and CL swabs were vortexed and 250 μL of the medium was used to extract the RNA using Trizol LS^®^ reagent (Ambion, Invitrogen Canada Inc., Burlington, ON, Canada) according to manufacturer’s guidelines. Total RNA was also extracted from the trachea, lung, kidney, reproductive tract (uterus, isthmus and magnum), cecal tonsils, and proventriculus using Trizol reagent (Ambion, Invitrogen Canada Inc., Burlington, ON, Canada) following the manufacturer’s protocol. The amount of the extracted RNA was quantified using Nanodrop 1000 spectrophotometer (Thermo Scientific, Wilmington, DE, USA) at 260 nm wavelength. The quality of the RNA was determined based on the 260/280 absorbance ratio. Approximately 1000 ng from swab samples and 2000 ng of tissue samples were used to synthesize cDNA using RT random primers (High capacity cDNA reverse transcriptase kit, Invitrogen Life Technologies, Carlsbad, CA, USA) according to manufacturer’s guidelines.

#### 4.6.2. IBV Genome Load Quantification by qRT-PCR

The qRT-PCR assay was carried out using a CFX 96-c1000 Thermocycler (Bio-Rad Laboratories, Mississauga, ON, Canada) to quantify the IBV genome loads in cDNA samples. The assay was performed using Fast SYBR^®^ Green Master Mix (Invitrogen, Burlington, ON, Canada) maintaining the final reaction volume at 20 µL. Each reaction volume consisted of 10 µL of SYBR Green master mix, 100 ng of respective samples, 0.5 µL of forward Fw-5′GACGGAGGACCTGATGGTAA-3′ and reverse Re-5′CCCTTCTTCTGCTGATCCTG-3′ specific primers targeting the conserved IBV *N* gene [60]. Thermal cycling conditions were initial denaturation at 95 °C for 20 seconds (s); followed by 40 cycles of final denaturation at 95 °C for 3 s, and annealing at 60 °C for 30 s, a melting curve analysis was conducted between 65 °C to 95 °C with an increment of 0.5 °C at every 5 s.

#### 4.6.3. Histology

The kidney samples fixed in 10% neutral buffered formalin were sent to the Histopathology Diagnostic Services Unit at the University of Calgary, Faculty of Veterinary Medicine for further processing, sectioning, and hematoxylin-eosin (H&E) staining.

#### 4.6.4. Enzyme-Linked Immunosorbent Assay (ELISA)

Anti-IBV Abs in serum samples (1:500 dilution) were quantified using IDEXX ELISA kit (IDEXX Laboratories, Westbrook, ME, USA) according to manufacturer’s guidelines. This ELISA kit does not differentiate different Ab isotypes. The absorbance values were taken at 650 nm using the SpectraMax M2 microplate reader (Molecular Devices, Sunnyvale, NS, Canada) and the titers were calculated according to the manufacturer’s instruction. Titers greater than 396 were considered positive for anti-IBV Abs.

#### 4.6.5. Immunofluorescent Assay

OCT preserved frozen tissues were cut into sections of 5 µm thickness, adhered to positively charged slides (VWR, Missisauga, ON, Canada), and preserved at −20 °C until stained for CD4^+^ T cells, CD8^+^ T cells and macrophages. On the day of immunostaining, the sections were air-dried for 20 min (min) at room temperature and fixed with cold acetone for 5 min. The tissues were blocked with 5% goat serum diluted in Trizma buffered saline (TBS) buffer (Trizma base: 2.42 g; NaCl: 8 g in 1 L of distilled water; pH 7.6) and the mouse monoclonal Ab specific for chicken macrophages/monocytes (KUL01, Southern Biotech, Birmingham, AL, USA) or CD8α (CT-8, Southern Biotech, Birmingham, Alabama, USA) were used in a 1:200 dilution in 5% goat serum. The secondary Ab, goat anti-mouse IgG (H + L) conjugated with Dylight^®^ 550 (red fluorescence; Bethyl Laboratories Inc., Montgomery, TX, USA) was used in 1:500 dilution in 5% goat serum. The sections were incubated with primary Ab for 30 min and with secondary Ab for 60 min.

Immunostaining of CD4^+^ T cells consists of an additional 15 min of blocking step with avidin and biotin (Vector Laboratories, Inc., Burlingame, CA, USA) before blocking with 5% goat serum in TBS for 30 min. Each step was followed by 3 min of washing twice with Tris-buffered saline containing 0.1% Tween 20 (TBST) and once with PBS. Following blocking, the sections were incubated with 1:200 dilution of mouse monoclonal Ab specific to chicken CD4^+^ T cells (CT-4, Southern Biotech, Birmingham, Alabama, USA) in blocking buffer and incubated for 30 min. Next, the tissues were incubated for 30 min with 1:250 dilution of biotinylated goat anti-mouse IgG (H + L; Southern Biotech, Birmingham, AL, USA) and 15:1000 dilution of DyLight^®^ 488 (green fluorescence) streptavidin (Vector Laboratories Inc., Burlingame, CA, USA), respectively. Each incubation step was followed by 5 min of washing with TBST buffer twice and once with PBS. Finally, the slides were mounted with 4′,6-diamidino-2-phenylindole dihydrochloride (DAPI; DAPI, Vector Laboratories Inc., Burlingame, CA, USA; Blue fluorescence) and cover slipped.

### 4.7. Data Analysis

The number of IBV genome copies in 100 ng of RNA was calculated based on a standard curve obtained by serial dilution of plasmids. For quantification of immune cells, 5 areas (x20 magnification) of each tissue with the highest signals were captured using an epifluorescent microscope (Olympus IX51, Center Valley, PA, USA) along with applicable nuclear stained (DAPI) areas. The density of the cells in each area was quantified using Image-J software (National Institute of Health, Bethesda, MD, USA).

The difference in egg production between the infected and control groups was analyzed using a Mann–Whitney U test. The Mann–Whitney U test was also used to compare the anti-IBV Ab responses in serum of the infected group at two time points, 6 and 10 dpi. IBV genome load in OP, CL swabs and tissues were compared using the Kruskal–Wallis test followed by Dunn’s multiple comparison. Differences of CD4^+^ T cells, CD8^+^ T cells, and macrophages at 6 and 10 dpi were detected using the Mann–Whitney U test. All the statistical analysis was performed in GraphPad Prism Software (GraphPad Prism 9 Software, San Diego, CA, USA).

## Figures and Tables

**Table 1:** Genome features of the Canadian 4/91 IBV isolate, IBV/Ck/Can/17-038913.

UTR or ORF	Nucleotide Position	Nucleotide Length	Amino Acid Length
5′UTR	1–528	528	-
1a	529–12,384	11,856	3951
1b	12,459–20,417	7959	2652
Spike	20,368–23,862	3495	1164
3a	23,862–24,035	174	57
3b	24,035–24,229	195	64
E (3c)	24,210–24,539	330	109
M	24,511–25,188	678	225
4b	25,189–25,473	285	94
4c	25,394–25,555	162	53
5a	25,539–25,736	198	65
5b	25,733–25,981	249	82
N	25,924–27,153	1230	409
3′UTR	27,154–27,472	319	-

**Table 2:** Nucleotide sequence identity (%) of the Canadian 4/91 IBV isolate, IBV/Ck/Can/17-038913 compared to full-length sequences of representative IBV strains.

IBV Strain-Database Accession No.	Genome	1a	1b	Spike	3a	3b	E (3c)	M	4b	4c	5a	5b	N
4/91 vaccine-KF377577	90.7	88.5	93.3	96.6	93.7	89.7	83.0	92.4	88.1	83.0	84.3	96.4	90.9
Arkansas Vaccine-GQ504721	90.5	90.3	94.1	83.5	93.7	90.3	86.7	90.6	91.9	85.4	88.4	97.6	96.4
B1648-KR231009	90.4	91.4	92.4	85.3	93.7	88.2	*72.4*	91.6	91.6	83.6	86.9	95.2	93.3
California 99-AY514485	90.8	91.0	94.4	82.9	**94.8**	89.2	90.6	92.9	91.9	84.8	88.4	98.0	94.7
Ck/Aus/N1/08-KU556807	85.5	86.5	90.6	70.9	87.4	86.7	83.8	87.0	86.0	80.1	83.3	*91.6*	88.5
ck/CH/LSD/110857-KP118885	91.1	89.1	93.3	**96.7**	93.7	89.7	83.0	92.4	88.1	83.0	84.3	96.4	90.8
CK/SWE/0658946/10-JQ088078	88.3	89.7	91.0	81.0	84.5	*77.4*	84.7	*86.1*	91.9	88.3	86.9	94.4	88.9
Conn46 1996-FJ904716	91.7	92.5	94.5	83.7	**94.8**	89.2	86.4	90.6	91.9	85.4	88.4	**98.4**	**96.7**
Delaware 072-GU393332	87.6	89.3	92.6	66.8	91.4	90.3	90.6	97.2	85.3	78.9	87.4	**98.4**	95.1
γCoV/Ck/Poland/255/1997-MK581204	89.9	88.3	92.6	93.0	93.1	89.2	73.3	91.5	90.5	82.5	84.8	96.4	89.5
IBV_SES_15SK-02-MH539772	90.8	90.0	92.5	83.6	86.8	**93.3**	**97.9**	**99.0**	**97.9**	**98.1**	**97.5**	**98.4**	93.2
IBV/Ck/Can/17-036989-MN512435	90.3	90.0	**94.6**	81.0	**94.8**	89.2	91.5	96.2	92.6	85.4	88.4	**98.4**	95.7
IBV/Ck/EG/CU/4/2014-KY805846	87.3	86.7	90.4	82.4	90.8	88.7	73.3	90.9	88.4	78.4	84.3	95.6	91.6
IBVUkr27-11-KJ135013	90.9	89.1	93.3	95.1	93.1	88.7	76.7	90.9	90.9	83.6	85.9	97.2	92.7
ITA/90254/2005-FN430414	89.2	90.8	90.8	81.5	85.6	77.9	88.6	92.5	90.5	83.0	85.9	96.8	92.1
JMK-GU393338	**92.0**	93.0	93.2	83.3	93.7	90.3	87.6	91.7	91.9	84.8	88.4	97.2	95.5
KM91-JQ977698	90.0	91.5	92.6	82.1	79.6	82.6	87.3	90.7	93.0	86.0	87.4	97.2	93.7
Mass41 2006-FJ904713	91.3	92.1	93.8	84.1	**94.8**	89.2	85.5	90.5	91.9	85.4	88.4	**98.4**	**96.7**
NGA/A116E7/2006-FN430415	90.3	91.4	92.5	85.6	85.6	85.1	73.9	91.2	89.4	*76.6*	85.9	95.6	92.1
SAIBK-DQ288927	87.5	87.4	90.1	81.9	84.5	83.3	88.7	88.9	85.3	83.6	82.3	96.0	*86.8*
TCoV/IN-517/94-GQ427175	87.2	**93.5**	94.3	*48.3*	93.7	88.7	90.0	90.8	91.6	83.6	87.4	97.6	96.6
TW2575/98-DQ646405	*86.1*	*85.1*	89.5	81.7	87.9	89.2	88.8	89.1	*84.6*	81.3	81.3	96.4	89.9
YN-JF893452	86.8	87.3	*89.4*	81.9	*83.9*	82.1	89.3	89.2	86.3	83.6	*80.8*	96.0	87.5

Bold font indicates the highest, and italic font indicates the lowest, nucleotide sequence identity.

**Table 3:** Histopathological changes observed in the kidney of IBV/Ck/Can/17-038913 isolate infected and control hens (10 dpi).

Histopathological Finding	4/91 IBV Infected	Control
Dilation of collecting duct	6/6	1/6
Increased urate spherules	6/6	0/6
Mononuclear cell infiltration	2/6	0/6
Sloughed epithelial cells in the lumen of collecting duct	4/6	0/6
Hyper eosinophilic necrotic cellular debris	2/6	0/6

## Data Availability

The datasets used and/or analyzed within the frame of the study can be provided by the corresponding author upon reasonable request.

## Supplementary Materials

The following are available online at https://www.mdpi.com/article/10.3390/pathogens10050624/s1, Figure S1: Egg production following infection with IBV/Ck/Can/17-038913 isolate, Table S1: List of reference IBV sequences used in the S1 sequence analysis.
