# Supplementary material for: Molecular Characterization of 4/91 Infectious Bronchitis Virus Leading to Studies of Pathogenesis and Host Responses in Laying Hens
Source: Pathogens. 2021 May 19;10(5):624. doi: 10.3390/pathogens10050624 (PMC8161358; doi:10.3390/pathogens10050624)
Supplement: Supplementary file 1 [file pathogens-10-00624-s001.zip › pathogens-1207274-supplementary.pdf]

pathogens

MDPI

Article

Molecular Characterization of 4/91 Infectious Bronchitis Virus Leading to Studies of Pathogenesis and Host Responses in Laying Hens

Shahnas M. Najimudeen [1,†], Mohamed S. H. Hassan [1,2,†], Dayna Goldsmith [1], Davor Ojkic [3], Susan C. Cork [1], Martine Boulianne [4] and Mohamed Faizal Abdul-Careem [1,*]

[1] Department of Ecosystem and Public Health, Faculty of Veterinary Medicine, University of Calgary, Calgary, AB T2N 4N1, Canada; fathimashahnas.moham@ucalgary.ca (S.M.N.); msh.hassan@ucalgary.ca (M.S.H.H.); dayna.goldsmith@ucalgary.ca (D.G.); sccork@ucalgary.ca (S.C.C.)
[2] Department of Poultry Diseases, Faculty of Veterinary Medicine, Assiut University, Assiut 71515, Egypt
[3] Animal Health Laboratory, University of Guelph, Guelph, ON N1G 2W1, Canada; dojkic@uoguelph.ca
[4] Department of Clinical Sciences, Faculty of Veterinary Medicine, University of Montréal, St. Hyacinthe, QC, J2S 2M2, Canada; martine.boulianne@umontreal.ca
* Correspondence: faizal.abdulcareem@ucalgary.ca; Tel.: +1-403-220-4462; Fax: +1-403-210-974
† Equal contribution.

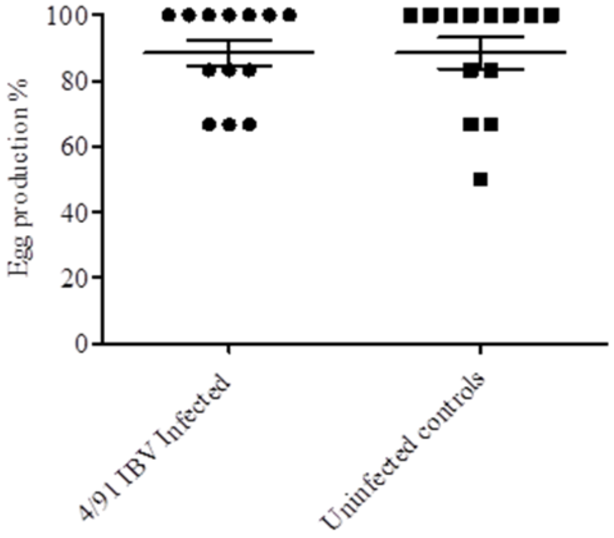

Supplementary Figure 1. Egg production following infection with Canadian 4/91 IBV isolate, IBV/Ck/Can/17-038913. The percent daily egg production by infected and uninfected chickens is shown. Mann Whitney U test was used to compare the difference in egg production each day (P > 0.05). The error bars represent the SEM.

Supplementary Table 1. List of reference IBV sequences used in the S1 sequence analysis

Reference sequence	GenBank Accession #	Origin
N1/08	JN176213	Australia
V18/91	U29521	Australia
N4/02	DQ059618	Australia
N5/03	DQ059619	Australia
N1/62	U29522	Australia
V2-02	DQ490215	Australia
B1648	X87238	Belgium
IBV/Brasil/351/1984	GU393339	Brazil
Qu_mv	AF349621	Canada
Qu16	AF349620	Canada
IBV_SES_15AB-01	MH539771	Canada
IBV/Ck/Can/18-049707	MN512438	Canada
48SD-96VI	KC577388	China
QXIBV	AF193423	China
CK/CH/LLN/111169	KF411040	China
Connecticut vaccine	KF696629	China
QS	JQ250818	China
SDW	DQ070840	China
SDIB781/2012	KF007209	China
TC07-2	GQ265948	China
SAIBK	DQ288927	China
CK/CH/JX/JA09-1	HQ018890	China
ck/CH/LHLJ/110664	JQ739299	China
GX2-98	AY251816	China
ck/CH/LSD/110857	JQ739375	China
4/91 vaccine	KF377577	China
CK/CH/LDL/97I	EF030995	China
CH GX NN1111	KC692317	China
Q1	AF286302	China
ck/CH/LGX/111119	KX640829	China
gammaCoV/ck/China/I0114/14	KY407556	China
gammaCoV/ck/China/I0118/14	KY407558	China
Eg/1265B/2012	KC533682	Egypt
It/497/02	DQ901377	Italy
IZO 28/86	KJ941019	Italy
IBV422	KF809791	India
V25	KF757451	India
Variant 1	AF093795	Israel
IS/1201	DQ400359	Israel
Variant 2	AF093796	Israel
JP8443	AY296745	Japan
K620/02	FJ807944	South Korea
SNU8067	JQ977697	South Korea
A	AF151953	New Zealand
D	AF151956	New Zealand
K43	AF151958	New Zealand

D274	X15832	The Netherlands
V1397	M21968	The Netherlands
D1466	M21971	The Netherlands
BL-56	AF352831	Mexico
Moroccan-G/83	EU914938	Morocco
NGA/N544/2006	FN182269	Nigeria
NGA/324/2006	FN182277	Nigeria
NGA/295/2006	FN182276	Nigeria
NER/28/2007	FN182272	Niger
RF/01/02	AJ441314	Russia
Spain/00/336	DQ386098	Spain
Spain/98/313	DQ064808	Spain
TP/64	AY606320	Taiwan
3381/06	GQ229245	Taiwan
UK/L-633/04	DQ901376	United Kingdom
UK/7/91	Z83975	United Kingdom
6/82	X04723	United Kingdom
FR-85131-85	AJ618985	United Kingdom
Beaudette	M95169	USA
M41	AY561711	USA
Conn46 1996	FJ904716	USA
Iowa97	GU393337	USA
Gray	L14069	USA
JMK	L14070	USA
PA/5344/98	AY789947	USA
Holte	L18988	USA
L905	JQ964070	USA
SE17	M99484	USA
ARK99	M99482	USA
ArkDPI	AF006624	USA
CAL99	DQ912831	USA
PA/Wolgemuth/98	AF305595	USA
CA/1737/04	EU925393	USA
DMV/5642/06	EU694402	USA
GA/13485/2013	KP085597	USA
GA/13384/2013	KM660635	USA
GA08	GU301925	USA
DE/072/92	U77298	USA
GA/13055/00	AF338719	USA
CU82792	AF317214	USA
